# Ictal asystole in epilepsy patients undergoing inpatient video-EEG monitoring

**Published:** 2014-10

**Authors:** Bashir S. Shihabuddin, Aline S. Herlopian, L. John Greenfield

**Affiliations:** *From the Department of Neurology, University of Arkansas for Medical Sciences, Arkansas, United States of America*

## Abstract

Ictal asystole (IA) is uncommonly diagnosed and has been implicated as a potential cause of sudden unexpected death in epilepsy. Sudden unexpected death in epilepsy is an increasingly recognizable condition and is more likely to occur in patients with medically intractable epilepsy and those suffering from convulsive epilepsy. We report 2 cases of recent onset of prolonged syncope and unrevealing cardiac work up. The inpatient video-EEG monitoring recorded left temporal ictal discharges followed by IA. Although the role of cardiac pacing is controversial in these patients, both patients had favorable outcome following cardiac pacemaker insertion. This report demonstrates the variability in IA pathophysiology and clinical manifestations. It also advocates that cardiac pacing might have a role in the management of IA.

Ictal asystole (IA) is an uncommon event that occurs in 0.1-0.4% of patients experiencing seizures during inpatient video-electroencephalography monitoring (VEEG).^1^ Ictal asystole is more likely to occur in patients with focal seizures originating from the temporal region.^2^ It has been implicated as a potential cause for sudden unexpected death in epilepsy patients (SUDEP) that carry an incidence of 0.09-9.3 per 1000 patient-years.^1^ Although controversial, cardiac pacing has been suggested as a possible preventive measure against IA and SUDEP.^3^ We report 2 cases of IA encountered at our epilepsy monitoring unit between June 2010 and October 2011, and their clinical outcomes after cardiac pacemaker insertions. These 2 cases highlight the importance in considering IA as a potentially fatal complication of epileptic seizures that might be circumvented by good seizure control, and in some cases cardiac pacing.

## Case Report

### Patient one

**Figure 1:**
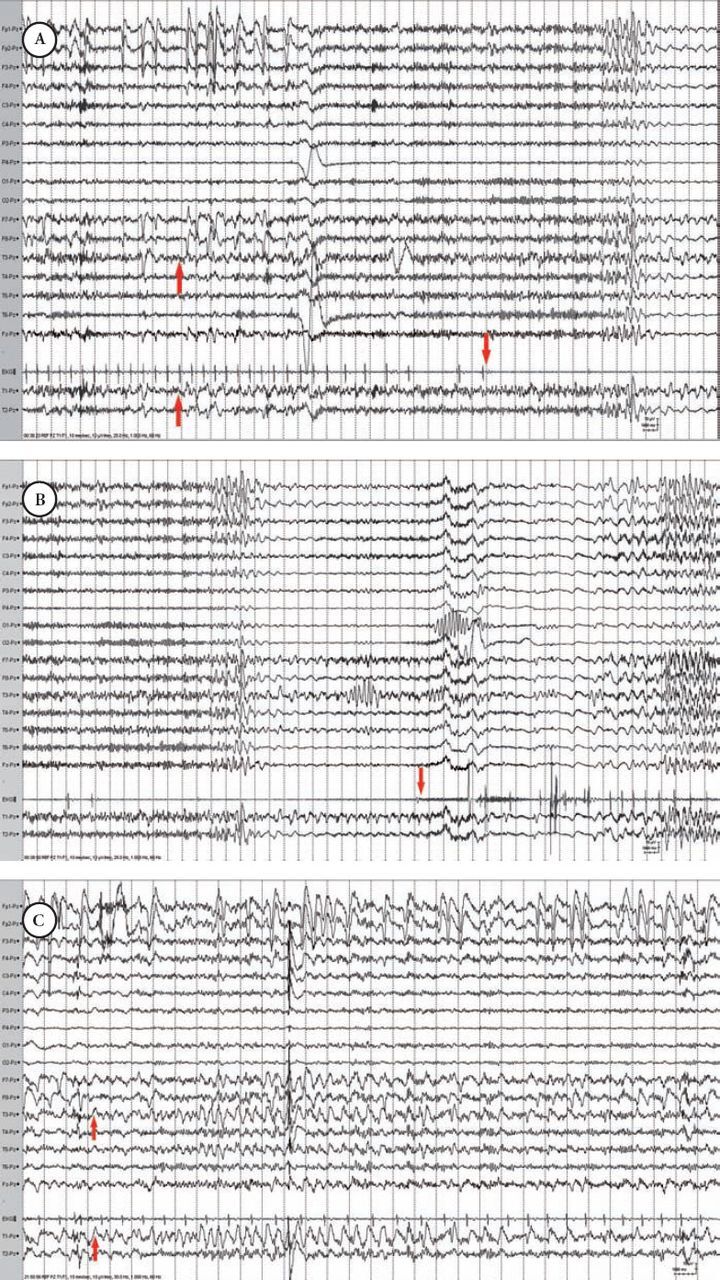
Ictal asystole and subclinical ictal discharge in patient one showing: **A**) Subtle left mid temporal ictal discharge onset (up arrows) followed by bradycardia and ictal asystole (down arrow). **B**) Termination of ictal asystole (arrow) followed by recovery of EEG activity. **C**) A subclinical ictal discharge of left mid temporal onset (arrows).

A 65-year-old woman presented with a history of convulsive seizures 20 years earlier that were fully controlled by phenytoin. One year prior to presentation she started experiencing recurrent syncope with lightheadedness rapidly progressing to loss of awareness and collapse. Afterwards, she was unresponsive and completely flaccid for approximately one minute. An extensive cardiac workup including tilt table test, Holter monitor, carotid Doppler’s and echocardiography were unrevealing. An MRI of the brain with and without contrast showed age related mild global cerebral atrophy. Phenytoin was changed to levetiracetam due to the concern that the syncope events might be related to epileptic seizures and she was referred for inpatient VEEG. Levetiracetam was discontinued during VEEG and 2 syncopal events were recorded. Both were associated with left mid temporal ictal discharges that were initially associated with bradycardia and progressed to asystole within 5 and 9 seconds after the ictal onset and lasted 22 and 29 seconds (**Figures 1A & 1B**). During the cardiac asystole she was unresponsive and the EEG showed a severe voltage attenuation followed by diffuse delta activity, which transitioned to faster frequencies as the cardiac rhythm recovered and she regained consciousness. In addition, the prolonged EEG recorded a localized subclinical ictal discharge of left mid temporal onset that was not associated with any ECG abnormalities (**Figure 1C**). The following day she underwent a cardiac pacemaker insertion. She was discharged on levetiracetam. Twenty-one months later she remained free of syncopal events, but reported rare and brief sensations of lightheadedness lasting for few seconds.

### Patient two

**Figure 2:**
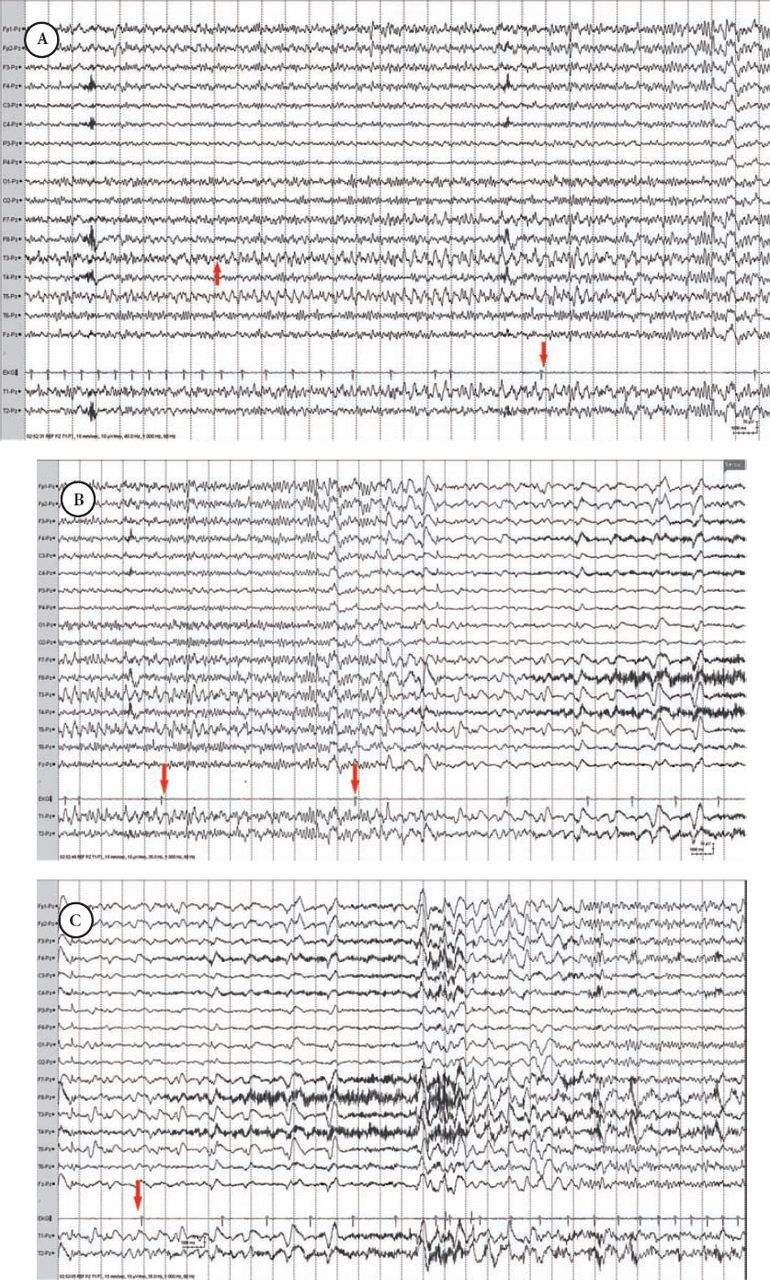
Ictal asystole in patient 2 showing: **A**) Left mid temporal ictal discharge onset (up arrow) followed by bradycardia and ictal asystole (down arrow). **B**) Ictal asystole (arrows) associated with EEG voltage attenuation. **C**) Termination of ictal asystole followed by recovery of the EEG activity (arrow).

A 64-year-old woman presented with a 3-year history of syncopal episodes occurring every 1-2 months. The clinical events were stereotypical. She would collapse to the ground without any warning and be flaccid and unresponsive for up to 20 minutes. She underwent an unrevealing cardiac work up, and an MRI of the brain showed mild cerebral atrophy. She was initially placed on levetiracetam for presumed seizures. Valproic acid was added when syncope recurred, and she was referred for inpatient VEEG. After both levetiracetam and valproic acid were discontinued. an ictal discharge was recorded from the left mid temporal area during sleep. Twelve seconds after the ictal onset she developed sinus bradycardia and 28 seconds later cardiac asystole occurred lasting for 9 seconds (**Figure 2**). The EEG during that period showed diffuse slowing and voltage attenuation. As the cardiac rhythm recovered, the EEG activity normalized. Following a Persantine nuclear imaging test and cardiac catheterization, cardiology opted against cardiac pacing and recommended good seizure control as a means to prevent cardiac arrhythmia. She was discharged on levetiracetam and valproic acid. However, 4 months later she experienced another prolonged syncope and a cardiac pacemaker was inserted. Four months following the procedure she had not experienced any further events suggestive of seizures or syncope.

## Discussion

Along with postictal cerebral suppression and respiratory compromise IA is implicated as a potential cause of SUDEP.^1^ It is very likely that all 3 causes are contributory during a seizure culminating in SUDEP. The Mortality in Epilepsy Monitoring Units Study (MORTEMUS)^4^ reported 16 SUDEP cases during inpatient VEEG. Fourteen of these deaths occurred at night. In all cases, SUDEP was triggered by a generalized tonic-clonic seizure followed by EEG suppression then central apnea and eventually severe bradycardia and asystole.^4^ Scheule et al^5^ proposed that IA is a self-limiting phenomenon as it results in cerebral hypoxia/anoxia, which hastens seizure termination while SUDEP is a consequence of complete cardiopulmonary arrest due to post-ictal suppression of cerebral activity. Moreover, SUDEP occurs primarily in patients with a long history of intractable symptomatic generalized epilepsy.^1^

Our 2 patients were of interest as both did not suffer from intractable epilepsy and both had focal seizures without secondary generalization. One patient had well controlled epilepsy for over 20 years before she started having focal seizures manifesting with ictal bradycardia and syncope. The other had a recent onset of focal seizures associated with ictal bradycardia and asystole. In both, the bradycardia and subsequent asystole was preceded by a left temporal focal ictal discharge. The insular cortex is essential in cardiovascular autonomic control. Seizure-induced activity in these autonomic centers may lead to excessive vagal tone and suppression of sympathetic tone resulting in ictal bradycardia and subsequent asystole.^1^ Although temporal lobe epilepsy is more frequently associated with ictal bradycardia and IA,^2^ there has been limited association between focal seizures and SUDEP. The authors could not find a documented case of SUDEP related exclusively to IA. In the MORTEMUS, all the 16 SUDEP cases and 7 out of the 9 near SUDEP cases had generalized tonic-clonic seizures.^4^ Both of our patients had left mid temporal ictal discharge onset that did not progress to generalization. This suggests a completely different physiological mechanism leading to IA in patients who experience generalized tonic-clonic seizures and those with IA starting shortly after a focal ictal onset. The poor understanding of this phenomenon translates into uncertainty regarding the management of IA. Is it better to implant a cardiac pacemaker or to optimize seizure control using anti-epileptic drugs (AEDs)? This question remains a topic of debate. The inability of AEDs to guarantee perfect control of seizures and the potential physical injury and potentially fatal outcome associated with prolonged asystole, argue for the use of an internal cardiac pacemaker. One of our patients had 21 months follow up post-pacemaker insertion, and she was free of any syncope events. Our second patient did not experience any syncope events following the cardiac pacemaker insertion, although had only 4 months of follow up. Similarly, Mosely et al^3^ reported a decrease in the number of falls following pacemaker insertion in patients with IA. A similar observation was also recently reported by Duplyakov et al.^6^ These data suggests that cardiac pacemaker insertion might reduce the risk of physical injury and possibly death in patients with IA. Another issue illustrated by these 2 cases is their atypical presentation for seizures. Both patients had events of collapse followed by flaccid unresponsiveness. They did not present with the typical clinical manifestations of temporal lobe epilepsy, and thus distinction between epilepsy and syncope was not feasible based on clinical semiology. This emphasizes the importance of VEEG in making an accurate diagnosis in patients with unexplained episodes of unresponsiveness and decreased muscle tone. It also suggests that ictal bradycardia or asystole should be considered if a patient is experiencing epileptic seizures accompanied by unresponsiveness and decreased muscle tone. This report and previous reports of IA and SUDEP during VEEG underscore the need for the continued presence of well-trained VEEG technicians in epilepsy monitoring units. If continuous visual monitoring is not possible, adjunctive use of cardiac telemetry maybe appropriate to ensure immediate detection of such arrhythmias. An additional safety measure during these studies is the possible use of dedicated respiratory monitoring equipment. Using all these safety measures in combination will ensure the immediate attention of the medical staff to such life threatening occurrences.

In conclusion, IA is an under recognized entity in patients with epilepsy. It should be included in the differential diagnosis of unexplained syncope, and should be suspected in epilepsy patients experiencing ictal bradycardia or flaccid unresponsiveness. Although IA is not uniformly fatal, cardiac pacemaker insertion in addition to AEDs, might diminish the severity of the clinical manifestation of seizures in certain patients with epilepsy and in some patients might safeguard against SUDEP.
